# Comparison of a new type of Dark Matter with the Milky Way and M31 grand rotation curves

**DOI:** 10.1038/s41598-024-74884-6

**Published:** 2024-10-15

**Authors:** Bruce M. Law

**Affiliations:** Department of Physics, Kansas State University, 116 Cardwell Hall, Manhattan, KS 66506-2601 USA

**Keywords:** Astronomy and planetary science, Physics

## Abstract

In the electron Born self-energy (eBse) model, free electrons are of finite-size and possess both a rest mass, *m*_*e*_, as well as, a Born mass, *m*_*e*_^*B*^ = 74,000 *m*_*e*_. The Born mass, which originates from the energy contained within the electric field that surrounds a finite-sized electron, serves as a Dark Matter (DM) particle in this theory (designated eBDM, electron Born Dark Matter). The equation of state for *m*_*e*_^*B*^ is *w = -1*, which implies that two Born masses experience a ***repulsive*** gravitational interaction. This repulsive gravitational interaction stabilizes the formation of a DM halo of *m*_*e*_^*B*^ particles, of typical halo size ~ 100 kpc, around a central mass *M* (e.g. a galaxy), where this gravitational stability arises from the competing attractive *M - m*_*e*_^*B*^ and repulsive *m*_*e*_^*B*^ - *m*_*e*_^*B*^ interactions. A solution of the linearized Poisson-Boltzmann equation, for this system, allows one to derive an expression for the rotational velocity *V*_*eBDM*_*(R)*, as a function of radius *R* from the galactic center. A composite model composed of rotational velocity contributions from the galactic bulge, galactic disk, as well as, *V*_*eBDM*_*(R)* is found to provide a good description of the Grand Rotation Curves for the Milky Way and M31 galaxies.

**Supplementary Information:**

The online version contains supplementary material available at 10.1038/s41598-024-74884-6.

## Introduction

There are numerous astronomical anomalies that indicate that the Universe is full of Dark Matter (DM)^1,2^. DM is a type of matter that is known to interact gravitationally but does not interact via the electromagnetic force, namely, DM does not emit, absorb, or reflect light. Light does, however, undergo gravitational lensing in the presence of a large DM mass where, for example, DM galactic halos can distort galactic shapes^3,4^, for weak lensing, or may even create multiple images of the same galaxy^3,4^, if strong lensing is present. DM halos are also thought to be the cause of anomalously high stellar^5–7^ velocities around a galactic center, as well as, high galactic cluster velocities observed in the Coma cluster^8^. These high velocities can only be explained via the presence of significant amounts of DM, over and above the visible baryonic matter^9^. Gravitational lensing has also enabled the observation of a physical separation between the DM and luminous baryonic component, in the collision of two galaxies, within the Bullet cluster^10,11^. The astronomical observations for DM, discussed thus far, have been on galactic length scales. DM is also required on cosmological length scales in order to understand the expansion history of the Universe. In the current cosmological paradigm, as encapsulated by the ΛCDM model, the Cosmic Microwave Background (CMB) anisotropy requires a significant DM component; this model indicates that the Universe is composed of approximately ~ 5% baryons (the ordinary matter), ~ 25% cold DM (CDM), and ~ 70% Dark Energy (DE)^12^. DE is a type of repulsive gravity that has been acting during the last ~ 7 billion years of the Universe’s expansion history and is thought to be caused by Einstein’s Cosmological constant (the Λ in the ΛCDM model).

Planets, Black Holes, and other non-luminous massive bodies have been ruled out as providing sufficient gravitational interaction to explain DM. CDM is thought to be a slow moving (non-relativisitic) particle that interacts gravitationally. But what is CDM? None of the particles in the Standard Model (SM) of particle physics are capable of explaining CDM. CDM is thought to be a new type of particle that lies outside the SM and, thus, if found, would form an extension to the SM. Certain CDM candidate particles (WIMPs, Weakly Interacting Massive Particles) are thought to interact not only gravitationally but also via a weak interaction. If CDM does indeed interact via a weak interaction then this would allow direct detection e.g. at the LHC or at other specialized DM detectors^1^. Thus far there have been no confirmed direct detections of any CDM candidate particles. For any DM candidate, extensive work would be required to prove that this DM particle does indeed exhibit the requisite properties in order to explain all of the DM observations listed above.

An alternative approach to explaining the DM observations is to assume that Newtonian gravity is modified at very low accelerations (of order $\sim {10^{ - 10}}m/{s^2}$)^13,14^. Such an assumption is able to explain many features attributed to DM, however, MOND (Modified Newtonian Dynamics) is an empirical observation in need of an explanation. Namely, what physical phenomenon gives rise to a modification of Newtonian gravity and, by implication, General Relativity at these low accelerations and why are the modifications of the form assumed in MOND?

Both the ΛCDM model, as well as, MOND exhibit challenges and shortcomings in providing an adequate explanation for the DM astronomical observations. The purpose of this publication is to introduce a new form of DM, arising from the electron Born self-energy (eBse) model, describe its properties, and compare this DM candidate with Grand Rotation Curves (GRC) around the Milky Way and M31 galaxies. GRC data consists of astronomical measurements of the rotational velocities of stars, satellite galaxies, and globular clusters, around the galactic center of spiral galaxies.

This publication is set out as follows. Earlier publications, by the author, considered the cosmological consequences of the eBse model with regards to DE^15,16^ and Cosmic Inflation (CI)^17^. The work on DE is of direct relevance to the current discussions and, therefore, is briefly summarized in the Section “Brief review of the eBse model”. The Section “Electrostatics near a charged sphere” recalls the well-known electrostatics of a neutral plasma, surrounding a central charge *Q*, where the physics involved has similarities to our DM discussions. The eBse Dark Matter model is described in Section “eBse Dark Matter model” where this model is extended to predict the velocity of stars and satellite galaxies around the galactic center as a function of radial distance *R*. The Section “Milky Way and M31 GRC analysis” applies the eBse DM model to an analysis of the GRC data for both the Milky Way and M31 galaxies. Finally the Section “Summary and discussion” provides a summary and discussion of our results.

## Brief review of the eBse model

For a flat, homogeneous, and isotropic Universe, General Relativity reduces to the much simpler Friedmann velocity equation given by^18^

1 $${H^2}={\left( {\frac{{\dot {a}}}{a}} \right)^2}=\frac{{8\pi G}}{{3{c^2}}}{\Pi _{tot}}$$.

This equation describes the cosmological expansion of the Universe where *H* is Hubble’s parameter, *a* is the scale factor, $\dot {a}$ is the scale factor velocity, ${\Pi _{tot}}$ is the total energy density of intergalactic space, *G* is Newton’s gravitational constant, and *c* is the speed of light in a vacuum. In the ΛCDM model^18^

2 $${\Pi _{tot}}=\frac{{{\Pi ^R}}}{{{a^4}}}+\frac{{{\Pi ^B}+{\Pi ^{DM}}}}{{{a^3}}}+\frac{{{\Pi ^{DE}}}}{{{a^{3(1+w)}}}},$$

which has energy density contributions from radiation (superscript *R*), baryons (*B*), DM, and DE. The equation of state for DE has been measured to be^19,20^

3 $$w=\frac{p}{{{\Pi ^{DE}}}} \approx - 1,$$

to a good approximation, where *p* is the pressure. Equation (3) implies that ${\Pi ^{DE}}$ is a form of “repulsive gravity”, that causes the Universe to accelerate when ${\Pi ^{DE}}$ is dominant. In Eq. (2) DM and DE are of unknown origin where ${\Pi ^{DM}}$ and ${\Pi ^{DE}}$ are treated as adjustable parameters.

The Universe’s expansion switches from a deceleration (when ${\Pi ^{DE}}$ is sub-dominant early in the Universe’s expansion history at $a<<1$) to an acceleration (when ${\Pi ^{DE}}$ is dominant at $a \simeq 1$) where this switch over from deceleration to acceleration occurs at a redshift of^21^

4 $${z_{da}}\sim 0.8.$$

The ΛCDM model is the currently accepted cosmological paradigm because this model accounts for the CMB anisotropy ~ 380,000 years after the Big Bang (BB), recent DE measurements ~ 14 billion years after the BB, as well as, numerous other astrophysical observations^18^. The ΛCDM model is supplemented by a period of exponential acceleration (Cosmic Inflation) that occurs prior to the ΛCDM phase. CI is required in order to explain the homogeneity and flatness of the CMB where thermal fluctuations $\delta T$ have been measured to be remarkable small ($\delta T/T\sim {10^{ - 5}}$)^18^. Unfortunately, in the currently accepted cosmological paradigm of CI and ΛCDM there are many unknowns. What caused CI? What is DE? What is DM?

In the eBse model the electron is assumed to be of finite size, with radius

5 $${R_e}=1.9 \times {10^{ - 20}}m,$$

where the value in Eq. (5) arises from the best experimental estimate deduced from the contact interaction energy between electrons and positrons measured at the LEP (large electron-positron collider)^22,23^. A finite, non-zero electron radius implies that the Born self-energy of this electron, corresponding to the energy contained in the electric field that surrounds this particle, is given by^24,25^

6 $$U_{e}^{B}=\frac{{{e^2}}}{{8\pi {\varepsilon _o}{R_e}}},$$

where *e* is the fundamental unit of charge and ${\varepsilon _o}$ is the vacuum permittivity. Gravity couples to all energies. Equation (2) is missing contributions from electric field energies in intergalactic space, thus, in the eBse model, Eq. (2) is replaced by^15,16^

7 $${\Pi _{tot}}=\frac{{{\Pi ^R}}}{{{a^4}}}+\frac{{{\Pi ^B}+{\Pi ^{DM}}+{\Pi ^{elec}}}}{{{a^3}}},$$

where ${\Pi ^{DE}}$ has been omitted from Eq. (7) because ${\Pi ^{elec}}$ quantitatively accounts for phenomena that previously had been attributed to DE. The dominant contribution to ${\Pi ^{elec}}$ arises from the electron Born self-energy, primarily because the electron radius is so small. Thus,

8 $${\Pi ^{elec}} \simeq {N_B}{\nu _e}(t)U_{e}^{B}$$

where ${N_B}$ is the baryon number density in intergalactic space today (at $z=0$), while ${\nu _e}(t)$ is the **time dependent** fractional ionization in the WHIM (Warm-Hot-Intergalactic-Medium). In the WHIM atomic hydrogen, in cosmic web voids, are attracted to cosmic filaments where they collisionally ionize into free electrons and protons at temperatures of ${10^5} - {10^7}K$^26^. This collisional ionization fraction changes with time *t*

9 $${\nu _e}(t)\sim {t^s}$$

as the cosmic web evolves. The exponent *s* determines whether the expansion of the Universe is accelerating ($s \geqslant 1$) or decelerating ($s<1$) in this model. As discussed previously^15,16^, Eqs. (8) and (9) quantitatively account for many features that are attributed to DE. In particular, in the eBse model, (i) ${\Pi ^{elec}}$ today (at $a=1$ and $z=0$) agrees with the magnitude of DE (namely, ${\Pi ^{DE}}={N_B}{\nu _e}(z=0)U_{e}^{B}$), (ii) the ratio of DE to B today (i.e. ${\Pi ^{DE}}/{\Pi ^B}$) can be quantitatively explained, (iii) the equation of state for eBse is identical to Eq. (3), and (iv) the deceleration-acceleration redshift (Eq. (4)) arises from the switch over from $s<1$ to $s \geqslant 1$.

There are additional cosmological consequences if electrons and positrons are of finite size. Due to their finite size there will be a maximum number density of ${n_{\hbox{max} }}=1/{(2{R_e})^3}$, for electrons and positrons, as no more than one electron (or positron) can be packed into a volume ${(2{R_e})^3}$. During the early Universe’s evolution, at sufficiently high temperatures ($>1MeV$), there is a chemical equilibrium between photons and electron-positron pair creation ($2\gamma \leftrightarrow {e^ - }+{e^+}$). However, this system falls out of chemical equilibrium above a glass transition temperature ${T_G}=1.06 \times {10^{17}}K$ because the electron and positron number density is constant at ${n_{\hbox{max} }}$ for $T \geqslant {T_G}$^17^. A constant number density necessarily also implies that the potential energy density is also constant. In cosmology a constant potential energy density gives rise to exponential acceleration, akin to Cosmic Inflation. Thus, the eBse model possesses elements which replicate the behavior of both DE^15,16^ and CI^17^ in a single model.

In the eBse model DE arises from the time dependent creation of free electrons, along with their associated Born self-energy $U_{e}^{B}$ (Eq. (6)), from the collisional ionization of atomic hydrogen in the WHIM at temperatures of ${10^5} - {10^7}K$. In the WHIM there will be other (residual) free electrons present, specifically, free electrons outside the temperature range ${10^5} - {10^7}K$. These residual free electrons will also possess an associated $U_{e}^{B}$ where their fractional ionization, represented by ${\nu _{e0}}$, is expected to be time independent. In the eBse model, it is natural to assume that these residual free electrons give rise to effects that should be associated with DM, namely^15,16^,

10 $${\Pi ^{DM}}={N_B}{\nu _{e0}}U_{e}^{B}\;{\text{with}}\;{\nu _{e0}}=0.127,$$

where the estimate for ${\nu _{e0}}$ arises from the Planck collaboration estimate for the ratio ${\Pi ^{DM}}/{\Pi ^B}$.

**Figure 1:**
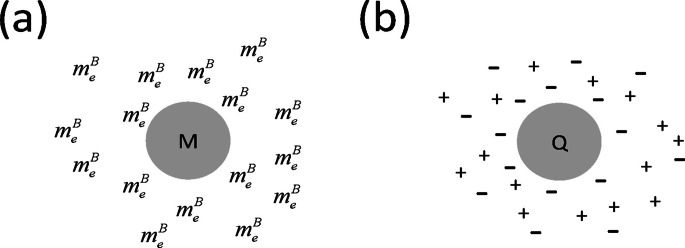
(**a**) Halo of Born masses around a central mass. (**b**) Plasma halo of positive and negative charges around a central (positive) charge .

To distinguish this new candidate eBse DM “particle” from other DM candidates (e.g. CDM) we will call it the electron Born DM (eBDM) candidate. The small value for ${R_e}$, in Eq. (5), gives rise to a surprisingly large electron Born mass of

11 $$m_{e}^{B}=U_{e}^{B}/{c^2} \approx 74,000\,{m_e} \approx 40\,{m_p},$$

where ${m_e}$ and ${m_p}$ are, respectively, the electron and proton rest masses. As the equation of state (Eq. (3)) holds for $m_{e}^{B}$, this implies that two $m_{e}^{B}$ masses are gravitationally repulsive (whereas a neutral, uncharged mass *M* and $m_{e}^{B}$ will be gravitationally attractive). There will be free electrons (forming a halo) around galaxies. An intuitive picture of the underlying physics that gives rise to galactic DM halos around galaxies, in the eBse model, is that there is a central mass *M* which is surrounded by a halo of Born masses $m_{e}^{B}$ around this central mass. The repulsive $m_{e}^{B} - m_{e}^{B}$ and attractive $M - m_{e}^{B}$ interactions stabilize the DM halo in this model (Fig. 1a). The physics for this situation is directly related to the formation and stability of a plasma “halo” that forms around a central (positive) charge *Q* (Fig. 1b). In this later case the halo stability arises from the fact that like charges repel, whereas, unlike charges attract.

There are some differences between the physics in Figs. 1a compared with Fig. 1b. In Fig. 1b one has electroneutrality and the number of positive charges (+) is balanced by the number of negative charges (-). By contrast, in Fig. 1a, there is only one type of repulsive mass, $m_e^B$, as the “counterbalancing” repulsive mass arising from the proton Born mass is much, much smaller ($m_p^B\approx9\times10^{-4}\;m_p$ using $R_p=8.5\times10^{-16}\;m$^27^ in Eq. (6)) and, therefore, can be neglected.

Any model, which is able to explain DM, will necessarily be an extension of the Standard Model of particle physics. A requirement for any extension of the SM is that it not create any conflicts with the SM. The eBse model is an extension of Quantum Electrodynamics (QED); QED forms part of the Standard Model. In QED the electron is assumed to be a point particle ($R_e\rightarrow0$) and, as a consequence, $U_e^B$ (Eq. (6)) is divergent. This divergence is“renormalized” away, in QED, by assuming that $U_e^B$ is subsumed or contained within *m*_*e*_. Unfortunately, the elimination of $U_e^B$ in QED implies that (non-local) energy is not conserved when considering the interaction between an electron and a positron^16^. Removal of $U_e^B$ also implies that QED is inconsistent with the treatment of ions in Soft Matter physics where the Born self-energy of ions is required to explain the solubility of ions in solution^24^. In the eBse model the electron is put on an identical footing to other ions, which possess both a normal rest mass, as well as, a Born self-energy while at the same time preserving conservation of energy. As discussed in the Supplementary Information in^17^ the value for *R*_*e*_, in Eq. (5), is sufficiently small that QED experiments continue to be consistent with QED theoretical predictions, thus, ensuring the integrity of the Standard Model.

## Electrostatics near a charged sphere

Before we consider the eBse mass contribution to the Dark Matter galactic halo, it is first useful to review the electrostatics of a plasma of protons and electrons that surround a single fixed test charge *Q*. Here we follow the presentation of Thorne and Blandford^28^ Chap. 20. The electrostatic potential $\phi (r)$ outside a charged particle satisfies Poisson’s equation.

12 $$\begin{gathered} {\nabla ^2}\phi = - \frac{\rho }{{{\varepsilon _o}}} \hfill \\ \quad \;\;= - \frac{{({n_p} - {n_e})e}}{{{\varepsilon _o}}} - \frac{Q}{{{\varepsilon _o}}}\delta (r), \hfill \\ \end{gathered}$$

where $\rho =\rho (r)$ is the charge density, ${n_i}(r)$ is the average number density of protons or electrons ($i=p,e$), while $\delta (r)$ is the Dirac delta function. In the linearized Poisson-Boltzmann approach, where it is assumed that $e\phi <<{k_B}T$, then

13 $${n_p}=\bar {n}\exp \left[ { - \frac{{e\phi }}{{{k_B}T}}} \right] \approx \bar {n}\left[ {1 - \frac{{e\phi }}{{{k_B}T}}} \right]$$

14 $${n_e}=\bar {n}\exp \left[ {+\frac{{e\phi }}{{{k_B}T}}} \right] \approx \bar {n}\left[ {1+\frac{{e\phi }}{{{k_B}T}}} \right]$$

with $\bar {n}$ the mean number density of electrons or protons averaged over a large volume, ${k_B}$ is Boltzmann’s constant, and *T* is temperature. In this approximation Eq. (12) reduces to

15 $${\nabla ^2}\phi =\frac{{2\phi }}{{{\lambda _D}^{2}}} - \frac{Q}{{{\varepsilon _o}}}\delta (r),$$

where the Debye screening length is given by

16 $${\lambda _D}={\left( {\frac{{{\varepsilon _o}{k_B}T}}{{\bar {n}{e^2}}}} \right)^{1/2}}.$$

Equation (15) has solution

17 $$\phi (r)=\frac{Q}{{4\pi {\varepsilon _o}r}}{e^{ - \sqrt 2 r/{\lambda _D}}}.$$

For later considerations it is useful to consider the Debye screening length ${\lambda _D}$ in interstellar and intergalactic media, where the characteristic values are listed in Table 1 below, which were obtained from Table 20.1 in^28^.

**Table 1:** Debye screening lengths.

Plasma	$\overline n\left(m^{-3}\right)$	$T(K)$	${\lambda _D}\;(m)$	${\lambda _G}\;(kpc)$*
Interstellar medium	${10^5}$	${10^4}$	$10$	$0.009$
Intergalactic medium	$0.1$	${10^6}$	${10^5}$	$91$

*1 kiloparsec (kpc) = 3.086 × 10^19^ m.

## eBse Dark Matter model

### Galactic halo

According to the previous section the Debye screening length, ${\lambda _D}$, sets the characteristic length scale over which the electron and proton plasma, surrounding a charge *Q*, decays away. If the analogy in Fig. 1a and b is correct then there should also exist a characteristic gravitational Debye screening length, ${\lambda _G}$, which would set the decay scale for the halo of Born masses, $m_{e}^{B}$, around a central mass *M*. The interrelationship between ${\lambda _D}$ and ${\lambda _G}$ is expected to be given by

18 $${\left( {\frac{{{\lambda _D}}}{{{\lambda _G}}}} \right)^2}=\frac{{{F_G}}}{{{F_e}}},$$

where

19 $${F_e}=\frac{{{e^2}}}{{4\pi {\varepsilon _o}{r^2}}}$$

and

20 $${F_G}=\frac{{G{{(m_{e}^{B})}^2}}}{{{r^2}}}.$$

Equations (18)–(20) imply that

21 $${\lambda _G}=2.8 \times {10^{16}}\;{\lambda _D}$$

which leads to the values for ${\lambda _G}$ given in Table 1. The intergalactic gravitational Debye length

22 $$\lambda _{G}^{g} \approx 91\;kpc\sim {R_h}$$

is very similar to typical DM halo radii, ${R_h}$. According to Table 1, the interstellar gravitational Debye length

23 $$\lambda _{G}^{s}( \approx 0.009\;kpc)<<\lambda _{G}^{g},$$

which will be relevant in later discussions. Equations (16) and (18) imply that

24 $${\lambda _G}={\left( {\frac{{{k_B}T}}{{4\pi \bar {n}G{{(m_{e}^{B})}^2}}}} \right)^{1/2}}$$.

The magnitudes of ${\lambda _D}$ and ${\lambda _G}$, in Table 1, indicate the vastly differing length scales over which electrostatics and “Born mass” gravitational effects are of relevance.

### Rotational velocities around a galactic center

The Poisson equation for gravitational fields is given by^29^

25 $${\nabla ^2}\phi =4\pi G\mu ,$$

where $\mu$ is the mass density. The ***sign*** difference between the “electrostatic” Poisson equation (Eq. (12)) and the “gravitational” Poisson equation (Eq. (25)) arises because gravity is always thought of as being attractive. If we consider a central mass *M* that is surrounded by a gaseous system of atomic hydrogen atoms, of mass ${m_H}$, then Eq. (25) becomes

26 $${\nabla ^2}\phi =4\pi G{n_H}{m_H}+GM\delta (r),$$

where the number density of hydrogen atoms is described by a Boltzmann distribution

27 $${n_H}={\bar {n}_H}\exp \left[ { - \frac{{{m_H}\phi }}{{{k_B}T}}} \right] \approx {\bar {n}_H}\left[ {1 - \frac{{{m_H}\phi }}{{{k_B}T}}} \right]$$.

Equations (26) and (27) reduce to

28 $${\nabla ^2}\phi =4\pi G{\bar {n}_H}{m_H} - \frac{\phi }{{{{\left( {\lambda _{G}^{H}} \right)}^2}}}+GM\delta (r),$$

where $\lambda _{G}^{H}$ represents the corresponding gravitational Debye length, but for atomic hydrogen (Eq. (24) with $m_{e}^{B}$ and $\bar {n}$ replaced by ${m_H}$ and ${\bar {n}_H}$, respectively). Upon comparison of Eq. (15) with Eq. (28), the key difference is the ***sign*** of the $\phi$ term on the right hand side. Equation (28) has solution

29 $$\phi (r)=\frac{{{k_B}T}}{{{m_H}}}+\frac{{GM}}{r}{e^{ - ir/\lambda _{G}^{H}}}$$

where the ${e^{ - ir/\lambda _{G}^{H}}}/r$ term represents a spherical wave, corresponding to gravitational collapse. Namely, as expected, a central mass *M* surrounded by atomic hydrogen is unstable. In Newtonian gravity, a description of gravitational collapse is obtained by combining Eq. (25) with the continuity and Euler equations, which leads to the gravitational Jeans instability^30^.

Equation (26) can be generalized to

30 $$\begin{gathered} {\nabla ^2}\phi =4\pi G[\mu +{n_H}{m_H}+{n_{He}}{m_{He}}+{n_p}{m_p}+{n_e}({m_e} - m_{e}^{B})]+GM\delta (r) \hfill \\ \quad \;\; \approx 4\pi G(\mu - {n_e}m_{e}^{B})+GM\delta (r) \hfill \\ \end{gathered}$$

which is more representative of the situation for galactic systems where, in the first line, a central mass *M* is surrounded by ordinary matter, with mass density $\mu =\mu (r)$, as well as, a gaseous cloud of atomic hydrogen, helium, and ionized hydrogen (free electrons and protons). In this first line the electron Born mass, $m_{e}^{B}$, makes an appearance but with a change in ***sign*** because, as mentioned before, two $m_{e}^{B}$ are repulsive. In the second line, in Eq. (30), only the dominant terms have been retained. In the following we consider a simplified model, with $\mu =0$, in order to examine how the eBse term influences stellar rotational velocities far from the galactic center (i.e. *M* represents a galactic mass, $M\sim ({10^9} - {10^{13}}){M_ \odot }$ where ${M_ \odot }$ is a solar mass).

In Eq. (30) the number density

31 $${n_e}=\bar {n}\exp \left[ { - \frac{{m_{e}^{B}\phi }}{{{k_B}T}}} \right] \approx \bar {n}\left[ {1 - \frac{{m_{e}^{B}\phi }}{{{k_B}T}}} \right]$$

which leads to the following linearized Poisson-Boltzmann equation

32 $${\nabla ^2}\phi = - 4\pi G\bar {n}m_{e}^{B}+\frac{\phi }{{{\lambda _G}^{2}}}+GM\delta (r)$$

where the “halo” gravitational Debye length, ${\lambda _G}$, is expected to lie between the interstellar and intergalactic limits, although nearer the later limit, namely, $\lambda _{G}^{s}<<{\lambda _G} \leqslant \lambda _{G}^{g}$. Equation (32) has solution

33 $$\phi (r)=\frac{{{k_B}T}}{{m_{e}^{B}}} - \frac{{GM}}{r}{e^{ - r/{\lambda _G}}}$$.

The gravitational potential energy for a mass $m_{e}^{B}$ at distance *r* from the galactic center is, therefore,

34 $$\psi _{e}^{B}(r)=m_{e}^{B}\phi (r)={k_B}T - \frac{{GMm_{e}^{B}}}{r}{e^{ - r/{\lambda _G}}}$$

which consists of a thermal “off-set” term and a screened gravitational potential energy. The screening arises from the presence of other $m_{e}^{B}$ masses which surround the central mass *M*.

From Eqs. (31) and (33) the electron Born mass density distribution is, therefore,

35 $$\begin{gathered} \mu _{e}^{B}(r)=\bar {n}m_{e}^{B}\left[ {1 - \frac{{m_{e}^{B}\phi (r)}}{{{k_B}T}}} \right] \hfill \\ \quad \;\;\;\;=\frac{1}{{4\pi }}\frac{M}{{r{\lambda _G}^{2}}}{e^{ - r/{\lambda _G}}}. \hfill \\ \end{gathered}$$

**Figure 2:**
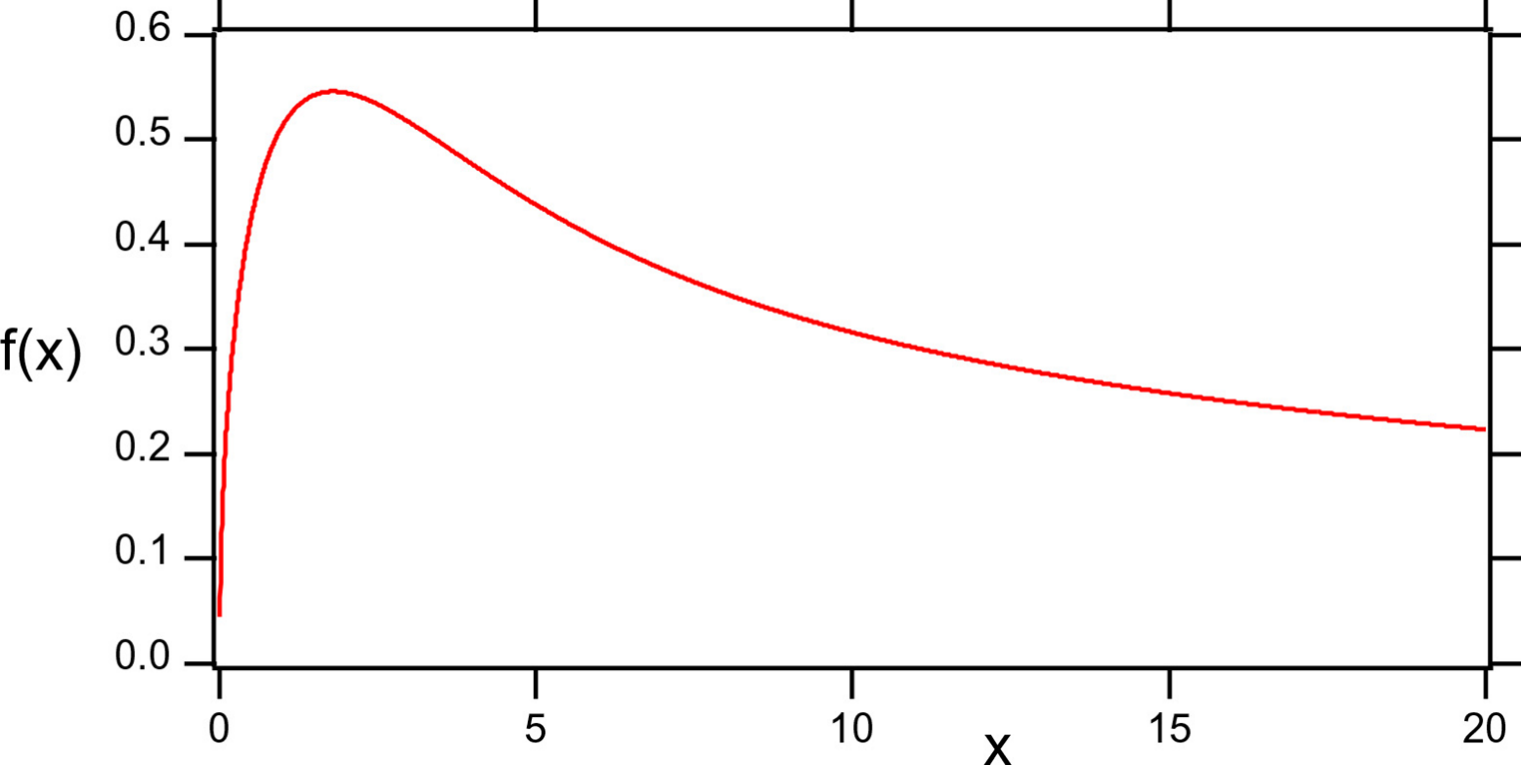
The universal function $f(x)$, Eq. (39), as a function of the dimensionless variable *x*.

The rotational velocity of a star about the galactic center, at radius *r*, is given by

36 $$V=\sqrt {\frac{{G{M_{Tot}}(r)}}{r}}$$.

where ${M_{Tot}}(r)$ is the total mass within radius *r*. In our later comparison with GRC galactic data we will already have including mass contributions from both the galactic bulge and disk, thus, we will only need to include contributions from DM in ${M_{Tot}}(r)$. Therefore

37 $$\begin{gathered} {M_{Tot}}(r)=\int {\mu _{e}^{B}(r)4\pi {r^2}dr} \hfill \\ \quad \;\;\;\quad \;={M_{eBDM}}\left[ {1 - \left( {1+\frac{r}{{{\lambda _G}}}} \right)\exp \left( { - \frac{r}{{{\lambda _G}}}} \right)} \right], \hfill \\ \end{gathered}$$

where ${M_{eBDM}}$ is the total DM mass that surrounds the galaxy. From Eqs. (36) and (37) the rotational velocity that arises from eBDM can therefore be written as

38 $${V_{eBDM}}=\sqrt {G{M_{eBDM}}/{\lambda _G}} f(x)$$

where the universal function

39 $$f(x)=\sqrt {\frac{{1 - (1+x)\exp ( - x)}}{x}}$$

with the dimensionless variable $x=r/{\lambda _G}$. The function $f(x)$, which is plotted in Fig. 2, is universal because its shape is independent of the particular value of ${\lambda _G}$. The decay length, ${\lambda _G}$, is a rescaling of the *r* axis (assuming constant ${\lambda _G}$) while both ${M_{eBDM}}$ and ${\lambda _G}$ influence the magnitude of the DM rotational velocity according to Eq. (38).

## Milky Way and M31 GRC analysis

Spiral galaxies typically consist of a central spherical bulge, of bulge radius ${R_b}\sim 2kpc$, upon which is superimposed a thin circular disk, of disk radius ${R_d}\sim 10kpc$ and disk thickness ${T_d}\sim 0.3kpc$. The bulge and disk, which are composed of dispersed stars, gas, and dust, are surrounded by a much larger and approximately spherical DM halo, of typical radius ${R_h}\sim 100kpc$. Stellar rotational velocities, about the galactic center, remain remarkable constant with velocities $V\sim 200 km/s$, for galactic distances in the range $R\sim 1 - 20kpc$. Only at very large distances, $R \geqslant 40kpc$, do the rotational velocities decrease appreciably^31^.

**Figure 3:**
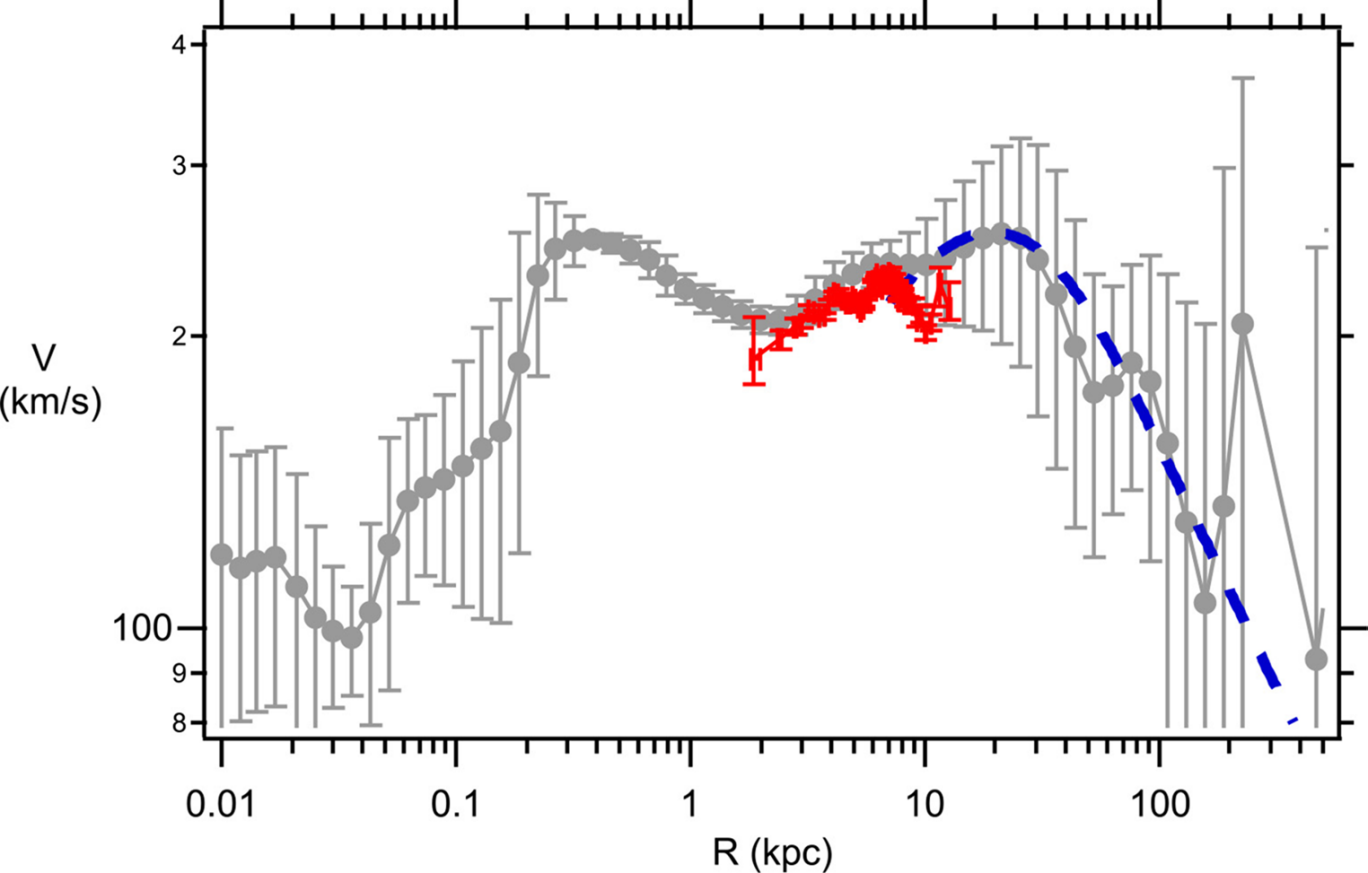
Milky Way GRC data from^31^ (gray data points including standard deviation errors). Data fit at large *R* using the eBDM model, Eqs. (38) and (39), with ${\lambda _G}=(10.9 \pm 1.2)kpc$ and ${M_{eBDM}}=(5.4 \pm 0.6) \times {10^{11}}{M_ \odot }$ (blue dashed line). Yu et al.^32^ data (red data points).

**Figure 4:**
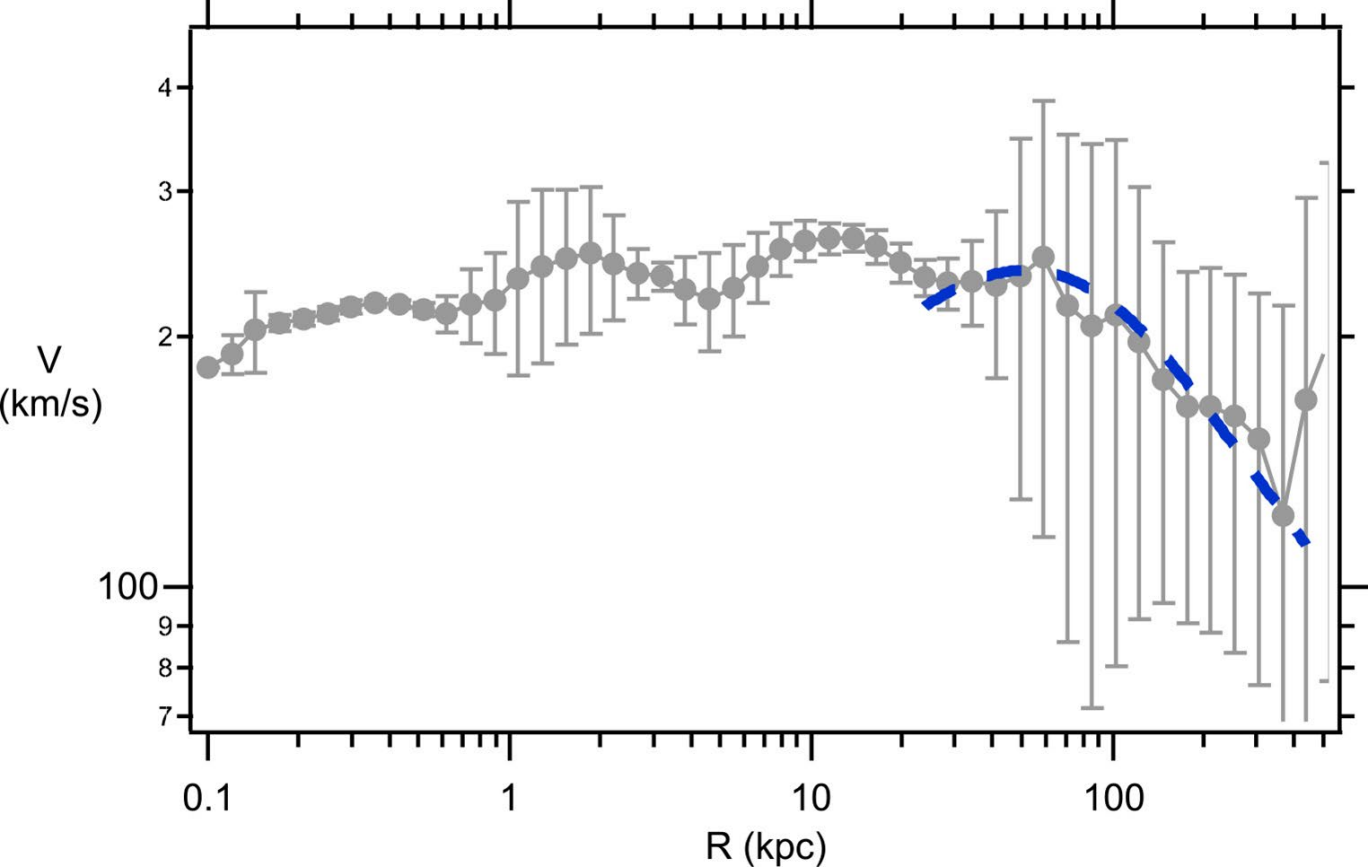
M31 GRC data from^31^ (gray data points including standard deviation errors). Data fit at large *R* using the eBDM model, Eqs. (38) and (39), with ${\lambda _G}=(28.6 \pm 2.6)kpc$ and ${M_{eBDM}}=(12.5 \pm 1.0) \times {10^{11}}{M_ \odot }$ (blue dashed line).

The Sofue GRC data^31^ (gray data points, with Standard Deviation errors) for the Milky Way and M31 galaxies are shown in Figs. 3 and 4, respectively. This GRC data is an amalgamation of stellar rotational velocities along with satellite galaxy and global cluster velocities around the respective galactic centers. As a test of the reliability of the Sofue MW GRC data the Yu et al.^32^ binned MW data is also shown on Fig. 3 (red data points). The Yu binned MW data represents the analysis of 2,706 measurements of gas tracers, star tracers, and masers that have been arranged into 52 radial bins with approximately 52 measurements in each bin. As can be seen from Fig. 3 the Sofue GRC data is very similar to the Yu binned data, except that the Sofue data is systematically shifted upwards by approximately $18km/s$. This upward shift arises from differences in the assumed solar rotational velocity used as a calibration. Sofue assumes a solar rotational velocity of ${V_0}=238 km/s$ at the Sun’s radial distance from the galactic center of ${R_0}=8.0kpc$, whereas, Yu assumes a value of ${V_0}=220 km/s$ at the same radial distance. The Sofue GRC data extends over a much, much larger range in *R* and, thus, is the most useful data set for ascertaining properties of the DM halo. Henceforth, any rotational velocities mentioned in this publication will be referring to the Sofue GRC data. For the MW (Fig. 3), the rotational velocities are approximately constant at $V\sim 230 km/s$ for galactic distances in the range $R\sim 0.3 - 40kpc$. Rotational velocities are observed to drop precipitously for *R* in the range $\sim 40 - 400kpc$. A similar behavior is observed for the M31 galaxy (Fig. 4). In this case rotational velocities are approximately constant at $V\sim 230 km/s$ for galactic distances in the range $R\sim 0.1 - 60kpc$. For $R \geqslant 60kpc$ there is a precipitous drop in *V* out to $R \approx 400kpc$. The influence of the DM halo is expected to play the greatest role at large *R*, therefore, as a preliminary test of the suitability for using the eBDM model to describe DM Eqs. (38) and (39) were fitted to the mean MW and M31 GRC data, at large *R*. The blue dashed lines, in Figs. 3 and 4, show the best fit where the values for the best fit parameters, ${\lambda _G}$ and ${M_{eBDM}}$, are listed in the figure captions. These preliminary comparisons indicate that the eBDM model may serve as a suitable model to describe DM.

### Modeling galactic rotational velocities

In modeling galactic rotational velocities the usual assumption is that the composite rotational velocity $V(R)$ is composed of three velocity contributions arising from the galactic bulge ${V_b}(R)$, galactic disk ${V_d}(R)$, and DM halo ${V_{DM}}(R)$ where^31^

40 $$V{(R)^2}={V_b}{(R)^2}+{V_d}{(R)^2}+{V_{DM}}{(R)^2}$$

and *R* is the galacto-centric distance.

The bulge contribution is frequently modeled using the de Vaucouleurs profile where the surface mass density^33^

41 $${\Sigma _b}(r)={\Sigma _{b0}}\exp [ - \kappa \{ {(r/{R_b})^{1/4}} - 1\} ]$$

with $\kappa =7.6695$ and ${\Sigma _{b0}}$ is the surface mass density at the half mass scale radius $R={R_b}$. The total bulge mass is then

42 $${M_b}=2\pi \int_{0}^{\infty } {r{\Sigma _b}(r)dr=\eta {R_b}^{2}{\Sigma _{b0}}}$$

where $\eta =22.665$. The bulge contribution to the rotational velocity is given by

43 $${V_b}(R)=\sqrt {G{M_b}/{R_b}} B(X),$$

where

44 $$B(X)={\left[ { - \frac{4}{{{M_b}X}}\int_{{r=0}}^{R} {{r^2}} \int_{{x=r}}^{\infty } {\frac{{d{\Sigma _b}(x)}}{{dx}}} \frac{1}{{\sqrt {{x^2} - {r^2}} }}dx\;dr} \right]^{1/2}}$$

and $X=R/{R_b}$. It is readily shown, via a few changes of variables, that Eq. (44) is mathematically equivalent to

45 $$B(X)={\left[ {\frac{\kappa }{{\eta X}}\int_{{Z=0}}^{X} {\int_{{Y=1}}^{\infty } {\frac{{{Z^{5/4}}\exp [ - \kappa \{ {{(ZY)}^{1/4}} - 1\} ]}}{{{Y^{3/4}}\sqrt {{Y^2} - 1} }}dY\;dZ} } } \right]^{1/2}}$$.

We experienced difficulties calculating $B\left(X\right)$, in the form given in Eq. (44), using Mathematica 13.3.1.0. Therefore, in these calculations, $B\left(X\right)$ was calculated using Eq. (45). Our $B\left(X\right)$ agrees well with the function given in Fig. 3 of^34^, at both small and large *X*, however, there are differences near the peak of $B(X)$, which we attribute to differences in the numerical code used for calculating $B(X)$. A derivation of Eqs. (43)–(45), along with a graph of $B(X)$ (Fig. S1), is provided in the Supplementary Information.

The galactic disk is usually modeled using a thin exponential disk which gives rise to a rotation velocity given by^34^

46 $${V_d}(R)=\sqrt {G{M_d}/{R_d}} D(X),$$

where

47 $$D(X)=\frac{X}{{\sqrt 2 }}{\left[ {{I_0}\left( {\frac{X}{2}} \right){K_0}\left( {\frac{X}{2}} \right) - {I_1}\left( {\frac{X}{2}} \right){K_1}\left( {\frac{X}{2}} \right)} \right]^{1/2}},$$

$X=R/{R_d}$, ${R_d}$ is the scale radius, ${M_d}$ is the total mass within the disk, and ${I_i}$ and ${K_i}$ are modified Bessel functions.

In the ΛCDM model, the DM halo is frequently modeled using the NFW density profile^35^ which is given by^34^

48 $$\rho (r)={\rho _o}/[Y{(1+Y)^2}],$$

where $Y=R/h$, and ${\rho _o}$ and *h* are the representative density and scale radius, respectively. The enclosed mass, within radius *R*, is then

49 $${M_{NFW}}(R)=4\pi {\rho _o}{h^3}[\ln (1+Y) - Y/(1+Y)]$$

which gives a rotation velocity of

50 $${V_{NFW}}(R)=\sqrt {G{M_{NFW}}(R)/R}$$.

It should be noted that the NFW profile was not derived from any fundamental principles (i.e. it was not derived from the gravitational Poisson equation Eq. (25)). Instead the NFW profile arises from a guess for the best fit to gravitational simulation data for the galactic formation of DM halos.

In the chi-squared fitting procedure^36^, used herein, a model for the composite rotational velocity *V* is fitted to the GRC data, ${V_i}$, by minimizing

51 $${\chi ^2}={\sum\limits_{{i=1}}^{N} {\left( {\frac{{V - {V_i}}}{{{w_i}}}} \right)} ^2},$$

where ${w_i}$ is the weight for each ${V_i}$, and *N* is the total number of data points. The minimization occurs by adjusting various parameters, within the function *V*, until ${\chi ^2}$ is a minimum. If *V* is a good representation of the data ${V_i}$, such that ${V_i}$ is normally distributed about *V* (i.e. a Gaussian process), then upon taking ${w_i}$ as the standard deviation ${\sigma _i}$ then the expectation value for chi-squared should be^36^

52 $$\left\langle {{\chi ^2}} \right\rangle =\nu =N - {N_c},$$

where $\nu$ is the number of degrees of freedom, which is the difference between the number of data points *N* and the number of constraints ${N_c}$ (i.e. the number of parameters that are actually adjusted to obtain the best fit).

In the ΛCDM model, the GRC data is chi-squared least-squares fitted using Eqs. (40), (43), (46), and (50) with 6 adjustable parameters, ${M_b}$ and ${R_b}$ for the bulge, ${M_d}$ and ${R_d}$ for the disk, and ${\rho _o}$ and *h* for the DM halo using the NFW profile. In the eBDM model, the NFW profile for DM, is replaced by Eqs. (38) and (39). In fitting the GRC data the 6 adjustable parameters are now, ${M_b}$ and ${R_b}$ for the bulge, ${M_d}$ and ${R_d}$ for the disk, and ${M_{eBDM}}$ and ${\lambda _G}$ for the DM halo.

**Figure 5:**
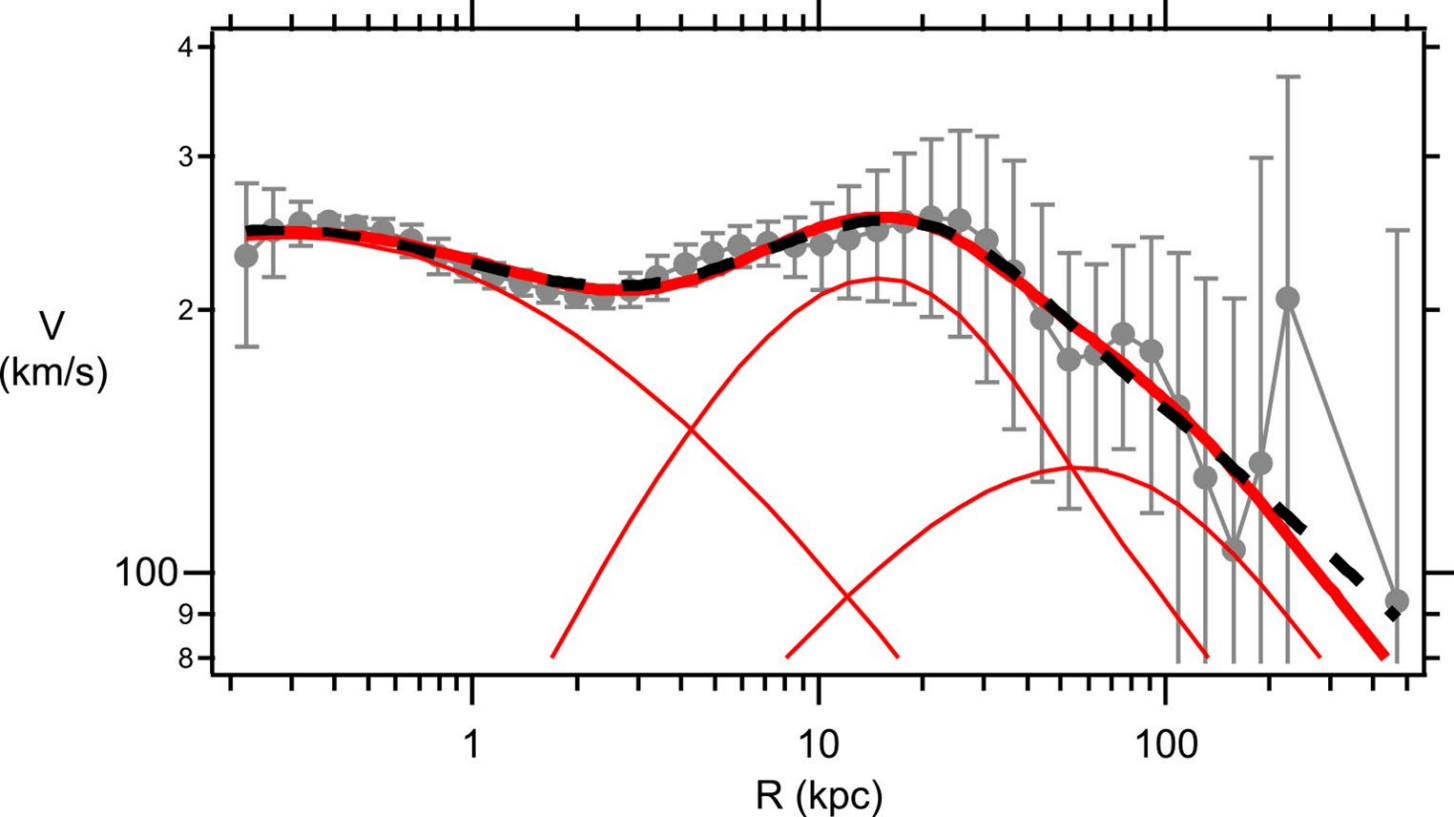
Milky Way GRC data (gray points with standard deviation errors). Best fit curve for ΛCDM Table 2 Trial 11 (black dashed line), eBDM Table 3 Trial 2 (red solid line). Three (lower) red solid curves, from left to right, ${V_b}$, ${V_d}$, and ${V_{eBDM}}$, which combine to eBDM Table 3 Trial 2. The anomalous datum point, at $R \approx 226\;kpc$, has been excluded from all fits.

For the Milky Way, the de Vaucouleurs profile does not fit the bulge at small *R*^37^, therefore, only GRC data in the range $0.2\;kpc \leqslant R \leqslant 500\;kpc$ is fitted. Additionally, the anomalous MW datum point, at $R \approx 226\;kpc$, has been excluded from all fits. The total number of data points fitted is $N=39$. Tables 2 and 3 provide ΛCDM and eBDM fitting data to the Milky Way GRC data, respectively, while various fitting curves along with GRC data is provided in Fig. 5. As discussed earlier (and in the Supplementary Information) discrepancies in the calculation of $B(X)$ imply that the Sofue best fit values (Table 2, Trial 1) do not provide a good fit to the GRC data in the current calculation. Following the recommendation of Sofue^34^ only the mean GRC data is fitted in the ${\chi ^2}$ fit (i.e. ${w_i}=1$). The Standard Deviation (SD) errors are excluded from the fit, as including these errors would de-emphasize data at large *R*. (The issue of the SD errors will be examined later in this discussion.) In Table 2, to obtain the best fit to the GRC mean values, we sequentially cycle through fitting the bulge, and then the disk, and then the NFW halo. In any particular trial only 2 parameters are free to be adjusted for the best fit; the other 4 parameters are left fixed at their most recent best fit values. For example, in Table 2 Trial 5, the following 4 parameters were left ***fixed*** at their most recent best fit values of ${R_d}=6.9,\;{M_d}=1.4,\;h=9.6,\;{\rho _o}=22$, while the 2 parameters ${R_b}$ and ${M_b}$ were allowed to vary to find the lowest ${\chi ^2}(=3748)$ value which resulted in ${R_b}=(0.70 \pm 0.08)\;kpc$ and ${M_b}=(0.19 \pm 0.02) \times {10^{11}}{M_ \odot }$. As one cycles through these various fits from Trial 1 to Trial 10 the ${\chi ^2}$ value steadily dropped from ${\chi ^2}=8679$ down to ${\chi ^2}=3534$, as the fit improves. One would hope that after 10 trials one would be close to the best fit minimum, corresponding to the lowest ${\chi ^2}$. However, by cycling through in the manner described one is, by construction, constraining (or forcing) the model to fit the data. As a test to see if Trial 10 is indeed located near the lowest ${\chi ^2}$ all 6 parameters were allowed to vary in Trial 11 (where the best fit values, in Trial 10, were used as the initial values in Trial 11). A disturbing feature of Trial 11 is that ${\rho _o}$ ballooned to ${\rho _o}=(40 \pm 41) \times {(10\;pc)^{ - 3}}{M_ \odot }$ where the error bar in ${\rho _o}$ is larger than its mean value. This issue could be indictive that the NFW profile is not a good description of the GRC MW data. The Igor Pro 9.0.5.1 ${\chi ^2}$ fitting procedure, used in this calculation, uses the Levenberg-Marquardt algorithm which assumes that the fitting function is a good description of the data and that the errors are normally distributed. The estimated error, for each fitting coefficient, is determined from the residuals.

**Table 2:** ΛCDM fit to Milky Way.

*Trial*	*R*_*b*_ (*kpc*)	*M*_*b*_ (10^11^*M*_⊙_)	*R*_*d*_ (*kpc*)	*M*_*d*_ (10^11^*M*_⊙_)	*h* (*kpc*)	*ρ*_*0*_ ((10 *pc*)^-3^*M*_⊙_)	χ^2^
1 (*Sofue*)	0.87	0.25	5.73	1.12	10.7	18.2	8679
2	0.68 ± 0.09	0.18 ± 0.02					4946
3			6.9 ± 0.4	1.4 ± 0.1			3882
4					9.6 ± 1.0	22 ± 5	3757
5	0.70 ± 0.08	0.19 ± 0.02					3748
6			7.3 ± 0.4	1.5 ± 0.1			3637
7					9.0 ± 0.9	26 ± 6	3595
8	0.72 ± 0.08	0.19 ± 0.02					3589
9			7.6 ± 0.4	1.5 ± 0.1			3553
10					8.6 ± 0.9	28 ± 6	3534
11	0.71 ± 0.22	0.19 ± 0.05	8.2 ± 1.1	1.6 ± 0.3	7.4 ± 3.0	40 ± 41	3483
12 (*SD*)	0.54 ± 0.24	0.16 ± 0.06	5.2 ± 1.7	1.1 ± 0.9	15 ± 31	10 ± 47	3.3

**Table 3:** eBDM fit to Milky Way.

*Trial*	*R*_*b*_ (*kpc*)	*M*_*b*_ (10^11^*M*_e_)	*R*_*d*_ (*kpc*)	*M*_*d*_ (10^11^*M*_e_)	*λ*_*G*_ (*kpc*)	*M*_*eBDM*_ (10^11^*M*_e_)	χ^2^
1 (*initial*)	1	1	5	1	40	2	
2	0.96 ± 0.15	0.25 ± 0.03	6.91 ± 0.74	1.9 ± 0.4	31 ± 10	4.07 ± 0.55	3795
3 (*SD*)	0.55 ± 0.15	0.17 ± 0.04	4.7 ± 1.6	1.0 ± 1.0	20 ± 21	4.7 ± 2.4	3.1

This ${\chi ^2}$ fitting issue, found for the ΛCDM fit, does not arise for the eBDM fit to the MW GRC data (Table 3). In this case, all 6 parameters were allowed to vary where the initial starting values are given in Table 3 Trial 1 and the resulting best fit values are given in Trial 2 of this table. In Table 3 Trial 2 all 6 error bars are less than their mean values, indicating that the eBDM model provides a good description of the data. Various best fit curves to the MW GRC data are shown in Fig. 5. Individual curves for ${V_b}$, ${V_d}$, and ${V_{eBDM}}$ from Table 3 Trial 2 are shown in Fig. 5 (red solid lines), which provides a sense for the range in *R* over which the bulge, disk, and eBDM contribute the most to the composite rotational velocity *V*. The disk-DM radius, ${R_{d/DM}}$, defined by

53 $${V_d}({R_{d/DM}})={V_{DM}}({R_{d/DM}})$$

determines whether galactic disk matter or DM dominates the composite rotation velocity. When $R>{R_{d/DM}}$ ($<{R_{d/DM}}$) DM (galactic disk material) dominates the composite rotational velocity *V*. From Fig. 5 ${R_{d/eBDM}}(MW)=53kpc$ for the eBDM model. DM is expected to be the dominant constituent of most galaxies. In the eBDM model, for the Milky Way, the percentage of DM is given by

54 $$D{M_{eBDM}}(MW)=\frac{{{M_{eBDM}}}}{{{M_b}+{M_d}+{M_{eBDM}}}} \times 100\% =65\%$$

which indeed indicates that the MW is dominated by DM, as expected. One can draw a number of interesting conclusions from Fig. 5, for the eBDM model. The individual ${V_b}$, ${V_d}$, and ${V_{eBDM}}$ curves, in this model, indicate that the observed flat rotation curve in the range $0.3kpc<R<20kpc$, for the MW, arises primarily from the distribution of matter in the galactic bulge and galactic disk. DM only becomes the dominant component for $R>{R_{d/eBDM}}(MW)=53kpc$ and, hence, plays a minor role in flattening the MW GRC curve (at $0.3kpc<R<20kpc$).

The MW is known to exhibit prominent dips in the rotational velocities at radii of $\sim 3kpc$ and $\sim 9kpc$^33^. These rotational dips are thought to arise from internal structure within the galactic disk. The model considered in this publication is axisymmetric, namely, the model only depends upon the galactic distance, *R*, and there is no angular dependence within the galactic disk. An axisymmetric exponential disk model, therefore, only captures the average physical features of galactic spiral arms and will be incapable of describing these rotational dips. Sofue, Honma, and Omodaka^33^ have examined what type of internal features, within the spiral arms, may be the cause for the rotational dips observed in the MW GRC data.

**Figure 6:**
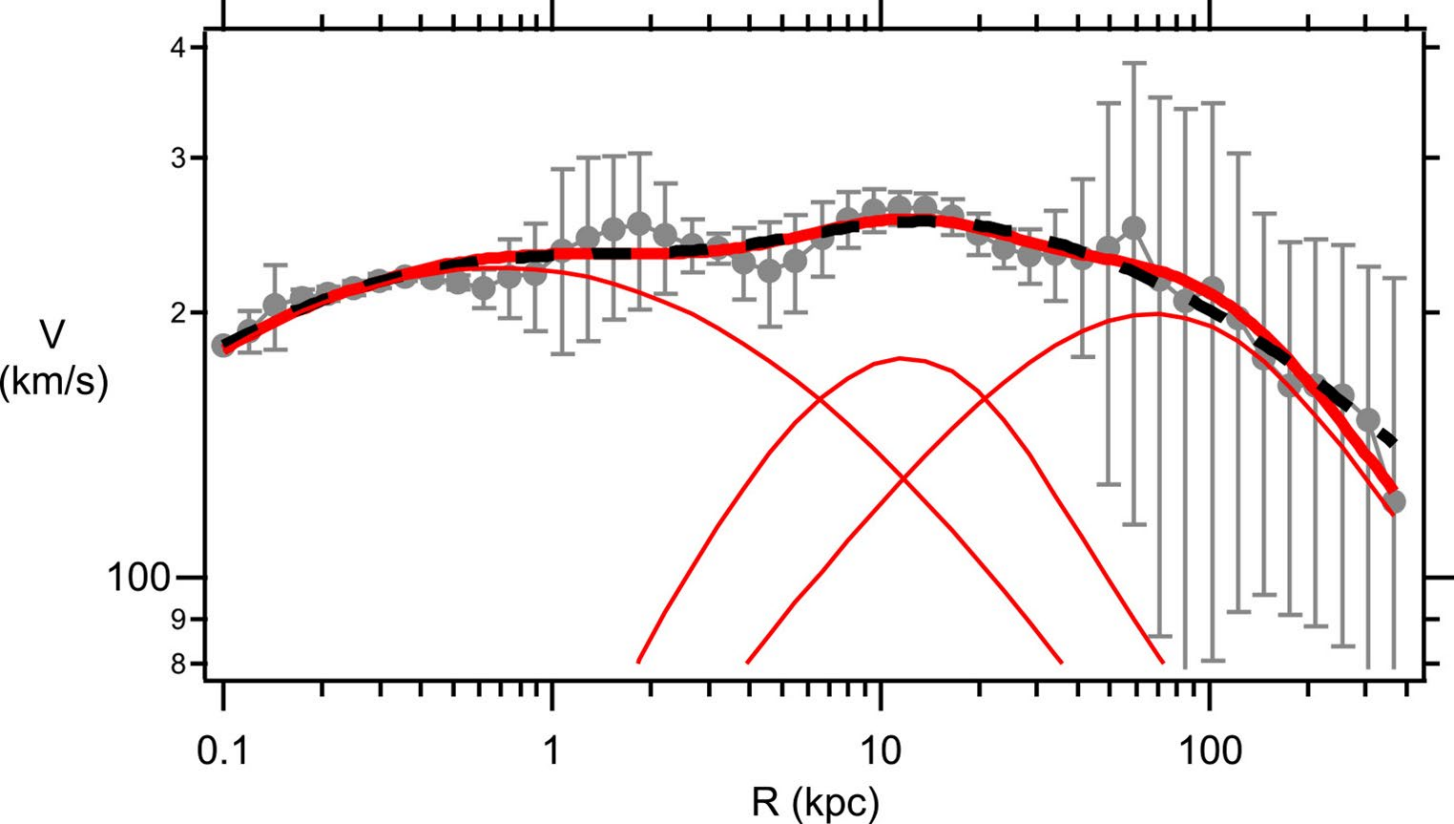
M31 GRC data (gray points with standard deviation errors). Best fit curve for ΛCDM Table 4 Trial 17 (black dashed line), eBDM Table 5 Trial 2 (red solid line). Three (lower) red solid curves, from left to right, ${V_b}$, ${V_d}$, and ${V_{eBDM}}$, which combine to eBDM Table 5 Trial 2.

This same ${\chi ^2}$ fitting scheme is applied to the M31 GRC data in Tables 4 and 5, and Fig. 6 for data in the range $0.1\;kpc \leqslant R \leqslant 400\;kpc$. The number of data points is $N=46$, in this case. After 16 trials (Table 4) one would hope that the lowest ${\chi ^2}$ had been determined for the ΛCDM model, however, upon allowing all 6 parameters to vary (Table 4, Trial 17) both ${M_d}$ and ${\rho _o}$ are found to have ballooned to ${M_d}=(0.24 \pm 0.42) \times {10^{11}}{M_ \odot }$ and ${\rho _o}=(25 \pm 22) \times {(10\;pc)^{ - 3}}{M_ \odot }$, in a similar manner to what was found for the MW GRC data. By contrast with this problematic ΛCDM behavior, the eBDM fit does not experience the same difficulties, and the best fitting parameters are readily fit without any of the parameters ballooning out of control (Table 5 Trial 2). Various best fit curves to the M31 GRC data are shown in Fig. 6. Individual curves for ${V_b}$, ${V_d}$, and ${V_{eBDM}}$ from Table 5 Trial 2 are shown in Fig. 6 (red solid lines), which provides a sense of the range in *R* over which the bulge, disk, and eBDM contribute the most to the composite rotational velocity *V*. The Dark Matter content for M31 is, in this case, $D{M_{eBDM}}(M31)=88\%$ where ${R_{d/eBDM}}(M31)=20kpc$. In the eBDM model the flat rotation curve for M31, which extends from $R\sim 0.2kpc$ through to $R\sim 70kpc$ (Fig. 6), is primarily dominated by the luminous galactic bulge and disk material for $R<{R_{d/eBDM}}(M31)=20kpc$ and DM only significantly contributes to flattening the rotation curves at $R>{R_{d/eBDM}}(M31)=20kpc$.

**Table 4:** ΛCDM fit to M31.

*Trial*	*R*_*b*_ (*kpc*)	*M*_*b*_ (10^11^*M*_⊙_)	*R*_*d*_ (*kpc*)	*M*_*d*_ (10^11^*M*_⊙_)	*h* (*kpc*)	*ρ*_*0*_ ((10 *pc*)^-3^*M*_⊙_)	χ^2^
1(*Sofue*)	1.35	0.35	5.28	1.26	34.6	2.23	15782
2	1.8 ± 0.2	0.39 ± 0.04					7527
3			5.8 ± 0.4	1.3 ± 0.1			7112
4					29 ± 3	3.0 ± 0.8	7665
5	1.9 ± 0.2	0.40 ± 0.04					6642
6			5.8 ± 0.4	1.2 ± 0.1			6512
7					26 ± 3	4.1 ± 0.9	6350
8	1.9 ± 0.2	0.41 ± 0.04					6232
9			5.9 ± 0.5	1.1 ± 0.1			5962
10					25 ± 3	4.8 ± 1.0	5850
11	2.0 ± 0.2	0.43 ± 0.04					5818
12			6.0 ± 0.5	1.08 ± 0.11			5629
13					23 ± 2	5.3 ± 1.1	5552
14	2.1 ± 0.2	0.45 ± 0.04					5520
15			6.1 ± 0.5	1.0 ± 0.1			5405
16					23 ± 2	5.8 ± 1.1	5347
17	1.95 ± 0.43	0.40 ± 0.11	5.1 ± 2.5	0.24 ± 0.42	13 ± 5	25 ± 22	4436
18 (*SD*)	1.25 ± 0.23	0.25 ± 0.05	4.2 ± 0.9	0.7 ± 0.6	17 ± 17	12 ± 26	6.8

**Table 5:** eBDM fit to M31.

*Trial*	*R*_*b*_ (*kpc*)	*M*_*b*_ (10^11^ M_e_)	*R*_*d*_ (*kpc*)	*M*_*d*_ (10^11^ M_e_)	*λ*_*G* _(*kpc*)	*M*_*eBDM*_ (10^11^*M*_e_)	χ^2^
1 (*initial*)	1	1	5	1	40	2	
2	2.4 ± 0.3	0.53 ± 0.08	5.6 ± 0.8	1.0 ± 0.2	38 ± 4	11.4 ± 0.7	4015
3 (*SD*)	1.4 ± 0.2	0.28 ± 0.05	4.5 ± 0.7	1.2 ± 0.3	40 ± 17	12 ± 6	6.9

The influence of the large SD errors, ${\sigma _i}$, upon the ${\chi ^2}$ fitting procedure is examined by setting the weight ${w_i}={\sigma _i}$. For this fit, there is much more “parameter space” within which each fitting parameter can vary. Table 2 Trial 12 shows the results for the ΛCDM fit to the MW GRC data where, in this fitting procedure, the Trial 10 results were used as the initial values. ${M_d}=1.1 \pm 0.9$, $h=15 \pm 31$, and ${\rho _o}=10 \pm 47$ have ballooned out of control. For the eBDM fit to the MW GRC data (Table 3 Trial 3) both ${M_d}=1.0 \pm 1.0$ and ${\lambda _G}=20 \pm 21$ have ballooned out of control. The ballooning is much worse for the ΛCDM model than for the eBDM model. Both fits have ${\chi ^2}\sim 3$, which is much less than the expectation value of $\left\langle {{\chi ^2}} \right\rangle =N - {N_c}=39 - 6=33$. This low ${\chi ^2}$ value is indicative that the SD errors are non-Gaussian and too large. Yu et al.^32^ also have found, in a separate analysis of MW data, that the SD errors were non-Gaussian.

The SD ${\chi ^2}$ fit to the M31 GRC data is given in Table 4 Trial 18 and Table 5 Trial 3 for, respectively, the ΛCDM and eBDM models. As was found for the MW GRC data, the eBDM model fits the M31 GRC data better than the ΛCDM model. The ${\chi ^2}\sim 6.9$ is much lower than the expectation value of $\left\langle {{\chi ^2}} \right\rangle =N - {N_C}=45 - 6=39$ indicating again that the SD errors are non-Gaussian and too large. (Note that in this fit $N=45$ because one datum point at ${R_i}=0.1kpc$ did not have a SD and, therefore, was omitted from the fitting procedure.)

## Summary and discussion

Any theory for DM must derive from the gravitational Poisson equation (Eq. (25)). If DM is a ***particle*** then this particle must obey the still more restrictive linearized Poisson-Boltzmann (lPB) equation. For a DM particle possessing an attractive gravitational interaction the lPB takes the form given in Eq. (28) and any galactic halo, formed from such particles, would be subjected to a gravitational instability and one would need to consider whether or not such a DM halo could remain stable over time periods of billions of years. By contrast, if the DM particle possesses a repulsive gravitational interaction the lPB takes the form given in Eq. (32) which would ensure the stability of the DM halo against gravitational collapse. Any theory for DM must also be able to explain the typical DM halo size, of order $\sim 100kpc$, as well as, provide a quantitative description of GRC data for the rotational velocities of stars, satellite galaxies, and globular clusters about the galactic center.

In this publication earlier work on the eBse model^15–17^ is extended to the DM galactic halo. The resultant eBDM profile possesses a repulsive gravitational interaction where the lPB gives rise to the DM rotational velocity ${V_{eBDM}}$ in Eqs. (38)–(39). Upon combining the de Vaucouleurs profile (Eqs. (43) and (45)) for the galactic bulge, and the exponential profile (Eqs. (46) and (47)) for the galactic disk, with the eBDM profile for the galactic DM halo, the resultant velocity (Eq. (40)) provides a good description of the GRC data for both the Milky Way (Table 3 and Fig. 5) and M31 (Table 5 and Fig. 6) galaxies. In comparison, the ΛCDM model, where the NFW profile (Eqs. (49)–(50)) is used to describe the DM halo (in place of the eBDM profile), the NFW profile does not describe the GRC data as well for both the Milky Way and M31 galaxies -- see Table 2, Trial 11 and Table 4, Trial 17 and the resultant discussion.

The eBDM model additionally provides natural explanations for a number of features associated with DM:

(i) The Born mass, $m_{e}^{B}$, is a feature of free electrons in the eBse model and will be present at all length scales and, hence, will influence galactic, as well as, cosmological astrophysical phenomena. $m_{e}^{B}$ only interacts gravitationally, and cannot be detected via any other means, which would explain why DM has not been detected directly thus far.
(ii) In the eBDM model the typical size of a DM galactic halo is expected to be of order the intergalactic gravitational Debye length $\lambda _{G}^{g} \approx 91kpc$ (Table 1).
(iii) In the ΛCDM model baryons and DM are expected to be uncorrelated, however, there is documented evidence (e.g. the Tully-Fisher relationship^38^) that there is a tight correlation between DM and baryons in galaxies. As the number of free electrons, in a galaxy, should be related to the number of baryons this suggests that, in the eBse model, there will be a tight correlation between eBDM and baryons in this model. An examination of this issue will be left to future research.
(iv) In the NFW profile, used in the ΛCDM model, there is a “cuspy halo” problem at small *R*^13,39^, namely, the DM density diverges as $\rho (R)\sim 1/R$ at small *R* (Eq. (48)). In reality DM is thought to be “flat”, rather than divergent, as $R \to 0$. In the eBDM model, at large $R\sim {R_h}\sim 100kpc$, the gravitational Debye length ${\lambda _G}$ is expected to be of order $\lambda _{G}^{g} \simeq 91kpc$. However, at small *R*, of order the bulge radius ${R_b}\sim 2kpc$, ${\lambda _G}$ is expected to cross-over to the interstellar gravitational Debye length $\lambda _{G}^{s} \approx 0.009kpc$. For very, very small $R<<{R_b}$ the physics will be controlled by the Coulomb interaction, between charged particles, rather than any gravitational interaction. Hence, in the limit $R \to 0$ we do not expect there to be a divergence in the DM density in the eBDM model. Note also, from Eq. (37), ${M_{Tot}}(R=0)=0$, in other words, in the eBDM model there is no divergent DM mass contribution at the origin.
(v) In the CDM scenario, there is a “missing satellites” problem where too many satellite galaxies are predicted to be orbiting the galactic center of large galaxies^40,41^. The eBDM model, along with associated baryon content, would need to be incorporated within galactic simulations before it is known what the eBDM model predicts.
(vi) DM is expected to induce structure formation where baryonic matter falls into the potential wells created by DM particles. The first line in Eq. (30) is valid in general, for any situation, where *M* represents a point mass at the origin. In the Section “Rotational velocities around a galactic center” we considered the situation where *M* was of order the galactic mass in order to derive the eBDM rotational velocity ${V_{eBDM}}(R)$ (Eqs. (38) and (39)). Alternatively, for a gaseous medium ($\mu =0$) and with $M=m_{e}^{B}$, Eq. (30) (when combined with the continuity and Euler equations) would describe both the repulsion between neighboring Born masses, as well as, the gravitational collapse of gaseous hydrogen, helium, and protons into the deep potential well created by each eBDM particle, due to the large Born mass ($m_{e}^{B} \approx 40{m_p}$, Eq. (11)).
(vii) The astronomical literature is replete with claims that “flat rotation curves provide evidence for DM”. The current analysis indicates that such claims should be treated with caution, especially if the claims are based upon rotational velocities that only extend out to galactic radii of $R\sim 10 - 20kpc$ (i.e. typical stellar rotational velocities). Figures 5 and 6 indicate that flat rotation curves out to $R\sim 10 - 20kpc$, for the Milky Way and M31 galaxies, are primarily determined by the mass distribution of baryonic matter in the galactic bulge and galactic disk. Only at much larger radii, $R>{R_{d/DM}}$ (Eq. (53)), does DM play a significant role in determining the rotational velocities around galactic centers. ${R_{d/eBDM}}(MW)=53kpc$ while ${R_{d/eBDM}}(M31)=20kpc$, therefore, the best evidence for DM from composite rotational velocities *V* arises at very large $R \approx 30 - 400kpc$ where the standard deviation errors in *V* are also unfortunately large. Prior analysis^31^, where the NFW profile was used to model the DM halo, would also seem to support this conclusion.
  To reiterate, in studying DM contributions from rotational velocities, it is very important to (a) accurately model the baryonic contributions from both the galactic bulge and galactic disk and (b) study rotational velocities at very large $R>{R_{d/DM}}$ where the DM contributions are greatest. Hence, studies of galactic Grand Rotation Curves (which include contributions from stars, satellite galaxies, and globular clusters) will provide more definitive information about DM than studies of galactic Rotation Curves (which only include contributions from stars). The close proximity of the Milky Way and M31 galaxies imply that their GRC data are likely to be very accurate and, hence, will serve as ideal candidates for the study of DM.
(viii) A MOND-like theory, which incorporated a Yukawa potential^42^, has recently been able to describe the GRC data for the Milky Way and M31 galaxies. ${V_{eBDM}}$, Eqs. (38)–(39), is similar to their apparent DM velocity, which would explain why these authors were able to obtain a good description of the GRC data. The Yukawa length, ${\lambda _Y}$ (where ${\lambda _Y}$ replaces ${\lambda _G}$ in Eq. (39)), that appears in such modified gravity theories is expected to be a ***universal constant***^43,44^. However, considerable variation in ${\lambda _Y}\sim 5 - 50kpc$ is observed from galaxy to galaxy^43,44^, thus, ${\lambda _Y}$ does not appear to be universal. (Note: these estimates for ${\lambda _Y}$were deduced by modeling the baryonic component using an exponential disk^43^ (i.e. no galactic bulge) at small galactic distances of $R\sim 0 - 25kpc$, hence, breaking both rules (a) and (b) in comment (vii) above). The expectation of a universal Yukawa length ${\lambda _Y}$, in modified gravity theories, should be contrasted with the eBDM model where the gravitational Debye length, ${\lambda _G}$, is expected to vary from galaxy to galaxy, depending upon the value of $T/\bar {n}$ in Eq. (24).

The eBse model is a “package deal”, namely, if this model is correct it is expected to describe DE^15,16^, DM, as well as, CI^17^. The construction of the eBse model is such that it is not possible to accept part of the model (e.g. DE) and reject the other two components (CI and DM) as this would lead to internal inconsistencies within the eBse model. As there is significant observational evidence for DM, the success or failure of the eBse model is therefore likely to hinge upon how well eBDM continues to describe these DM observations.

## Electronic supplementary material

Below is the link to the electronic supplementary material.

Supplementary Material 1

## Data Availability

All data generated or analyzed during this study are included in this published article.
