# Author Correction: Comparison of a new type of Dark Matter with the Milky Way and M31 grand rotation curves

**DOI:** 10.1038/s41598-024-78972-5

**Published:** 2024-12-05

**Authors:** Bruce M. Law

**Affiliations:** Department of Physics, Kansas State University, 116 Cardwell Hall, Manhattan, KS 66506-2601 USA

Correction to: *Scientific Reports* 10.1038/s41598-024-74884-6, published online 15 October 2024.

The original version of this Article contained errors in the headings of Tables 3 and 5, where the heading to Column 6 “$$\lambda_{G} (kpc)$$” was incorrectly given as “$$(kpc)$$” and in Column 7 “$$M_{eBDM} (10^{11} M_{e} )$$” was incorrectly given as *(*$$M)$$”.

The correct and incorrect Tables 3 and 5 appear below.

Incorrect:

**Table 3:** eBDM fit to Milky Way.

$Trial$	$R_{b} (kpc)$	$M_{b} (10^{11} M_{e} )$	$R_{d} (kpc)$	$M_{d} (10^{11} M_{e} )$	$(kpc)$	$M$	$\chi^{2}$
$1\;(initial)$	$1$	$1$	$5$	$1$	$40$	$2$	
$2$	$0.96 \pm 0.15$	$0.25 \pm 0.03$	$6.91 \pm 0.74$	$1.9 \pm 0.4$	$31 \pm 10$	$4.07 \pm 0.55$	$3795$
$3\;(SD)$	$0.55 \pm 0.15$	$0.17 \pm 0.04$	$4.7 \pm 1.6$	$1.0 \pm 1.0$	$20 \pm 21$	$4.7 \pm 2.4$	$3.1$

**Table 5:** eBDM fit to M31.

$Trial$	$R_{b} (kpc)$	$M_{b} (10^{11} M_{e} )$	$R_{d} (kpc)$	$M_{d} (10^{11} M_{e} )$	$(kpc)$	$M$	$\chi^{2}$
$1\;(initial)$	$1$	$1$	$5$	$1$	$40$	$2$	
$2$	$2.4 \pm 0.3$	$0.53 \pm 0.08$	$5.6 \pm 0.8$	$1.0 \pm 0.2$	$38 \pm 4$	$11.4 \pm 0.7$	$4015$
$3\,(SD)$	$1.4 \pm 0.2$	$0.28 \pm 0.05$	$4.5 \pm 0.7$	$1.2 \pm 0.3$	$40 \pm 17$	$12 \pm 6$	$6.9$

Correct:

**Table 3:** eBDM fit to Milky Way.

$Trial$	$R_{b} (kpc)$	$M_{b} (10^{11} M_{e} )$	$R_{d} (kpc)$	$M_{d} (10^{11} M_{e} )$	$\lambda_{G} (kpc)$	$M_{eBDM} (10^{11} M_{e} )$	$\chi^{2}$
$1\;(initial)$	$1$	$1$	$5$	$1$	$40$	$2$	
$2$	$0.96 \pm 0.15$	$0.25 \pm 0.03$	$6.91 \pm 0.74$	$1.9 \pm 0.4$	$31 \pm 10$	$4.07 \pm 0.55$	$3795$
$3\;(SD)$	$0.55 \pm 0.15$	$0.17 \pm 0.04$	$4.7 \pm 1.6$	$1.0 \pm 1.0$	$20 \pm 21$	$4.7 \pm 2.4$	$3.1$

**Table 5:** eBDM fit to M31.

$Trial$	$R_{b} (kpc)$	$M_{b} (10^{11} M_{e} )$	$R_{d} (kpc)$	$M_{d} (10^{11} M_{e} )$	$\lambda_{G} (kpc)$	$M_{eBDM} (10^{11} M_{e} )$	$\chi^{2}$
$1\;(initial)$	$1$	$1$	$5$	$1$	$40$	$2$	
$2$	$2.4 \pm 0.3$	$0.53 \pm 0.08$	$5.6 \pm 0.8$	$1.0 \pm 0.2$	$38 \pm 4$	$11.4 \pm 0.7$	$4015$
$3\,(SD)$	$1.4 \pm 0.2$	$0.28 \pm 0.05$	$4.5 \pm 0.7$	$1.2 \pm 0.3$	$40 \pm 17$	$12 \pm 6$	$6.9$

The original Article has been corrected.

