# Supplementary material for: Comparison of a new type of Dark Matter with the Milky Way and M31 grand rotation curves
Source: Sci Rep. 2024 Oct 15;14:24090. doi: 10.1038/s41598-024-74884-6 (PMC11480391; doi:10.1038/s41598-024-74884-6)
Supplement: Supplementary file 1 — Supplementary Material 1 [file 41598_2024_74884_MOESM1_ESM.docx]

Supplementary Information

Comparison of a new type of Dark Matter with the Milky Way and M31 grand rotation curves

Bruce M. Law^*^

*Department of Physics*

*116 Cardwell Hall*

*Kansas State University*

*Manhattan, KS 66506-2601*

*bmlaw@phys.ksu.edu

**Derivation of Eqs. (43) - (45) in the main text.**

Specifically,

$V_{b}(R)=\sqrt{GM_{b}/R_{b}}B(X)$, (43)

$B(X)=\left[-\frac{4}{M_{b}X}\int_{r=0}^{R} r^{2}\int_{x=r}^{\infty} \frac{d\Sigma_{b}(x)}{dx}\frac{1}{\sqrt{x^{2}-r^{2}}}dx dr\right]^{1/2}$ , (44)

where $X=R/R_{b}$. Alternatively, $B(X)$ can be shown to be mathematically equivalent to

$B(X)=\left[\frac{\kappa}{\eta X}\int_{Z=0}^{X} \int_{Y=1}^{\infty} \frac{Z^{5/4}\exp[-\kappa\{(ZY)^{1/4}-1\}]}{Y^{3/4}\sqrt{Y^{2}-1}}dY dZ\right]^{1/2}$. (45)

In the de Vaucouleurs profile the surface mass density is given by [[1](#_ENREF_1)]

$\Sigma_{b}(x)=\Sigma_{b0}\exp\left[-\kappa\left\{\left(x/\mathrm{R}_{b}\right)^{1/4}-1\right\}\right]$ , (S1)

therefore,

$\frac{d\Sigma_{b}(x)}{dx}=-\frac{\kappa\Sigma_{b0}}{4R_{b}}\frac{\exp\left[-\kappa\left\{\left(x/R_{b}\right)^{1/4}-1\right\}\right]}{\left(x/R_{b}\right)^{3/4}}$. (S2)

The volume mass density at radius $r$ is given by [[1](#_ENREF_1)]

$\rho(r)=-\frac{1}{\pi}\int_{x=r}^{\infty} \frac{d\Sigma_{b}(x)}{dx}\frac{1}{\sqrt{x^{2}-r^{2}}}dx$, (S3)

therefore, upon using Eq. (S2) and the change of variable $Y=x/r, dY=dx/r$

$\rho(r)=\frac{\kappa\Sigma_{b0}}{4\pi R_{b}}\int_{Y=1}^{\infty} \frac{\exp\left[-\kappa\left\{\left(rY/R_{b}\right)^{1/4}-1\right\}\right]}{\left(rY/R_{b}\right)^{3/4}\sqrt{Y^{2}-1}}dY$. (S4)

As the differential mass at radius $r$ is given by

$dM(r)=\rho(r) 4\pi r^{2}dr$, (S5)

therefore, the total integrated mass at galactic radius $R$ is

$M_{b}(R)=\int_{r=0}^{R} 4\pi r^{2}\rho(r)dr$. (S6)

Now using the change of variable $Z=r/R_{b}, dZ=dr/R_{b}$ we obtain

$M_{b}(R)=\int_{Z=0}^{R/R_{b}} \kappa\Sigma_{b0}R_{b}^{2}Z^{2}\int_{Y=1}^{\infty} \frac{\exp\left[-\kappa\left\{\left(ZY\right)^{1/4}-1\right\}\right]}{\left(ZY\right)^{3/4}\sqrt{Y^{2}-1}}dY dZ$. (S7)

Additionally, the total bulge mass [[1](#_ENREF_1)]

$M_{b}=\eta\Sigma_{b0}R_{b}^{2}$ (S8)

and the rotational velocity, from the bulge, at $R$

$V_{b}(R)=\sqrt{GM_{b}(R)/R}$ . (S9)

One can readily show, using Eqs. (S3), (S6), (S8), and (S9), that

$V_{b}(R)=\sqrt{GM_{b}/R_{b}}B(X)$ (S10)

where $X=R/R_{b}$ and

$B(X)=\left[-\frac{4}{M_{b}X}\int_{r=0}^{R} r^{2}\int_{x=r}^{\infty} \frac{d\Sigma_{b}(x)}{dx}\frac{1}{\sqrt{x^{2}-r^{2}}}dx dr\right]^{1/2}$. (S11)

Alternatively, Eqs. (S7) - (S9) gives rise to the equivalent expression

$B(X)=\left[\frac{\kappa}{\eta X}\int_{Z=0}^{X} \int_{Y=1}^{\infty} \frac{Z^{5/4}\exp[-\kappa\{(ZY)^{1/4}-1\}]}{Y^{3/4}\sqrt{Y^{2}-1}}dY dZ\right]^{1/2}$ (S12)

where $\kappa=7.6695$ and $\eta=22.665$.

The function $B(X)$ (Eq. (S12)), calculated using Mathematica 13.3.1.0, is shown in Fig. S1 (red solid line). Sofue [[2](#_ENREF_2)] also has evaluated $B(X)$, but using Eq. (S11). His evaluation for this function is shown as the dotted line in Fig. S1, which we obtained via a manual determination using his Fig. 3 in this reference. (For unknown reasons we experienced difficulties using Eq. (S11) to evaluate $B(X)$ in Mathematica.) The two evaluations of $B(X)$ are very similar except near the peak of this function; we assume that the differences between these two evaluations arise from differences in the numerical evaluation procedure. The peak of $B(X)$, occurs near $X=0.3$. We find $B(X=0.3)=0.7129$ which should be contrasted with the Sofue value of $B_{Sofue}(X=0.3)\simeq0.66$. This difference in the evaluation of $B(X)$ plays a role in the calculations in this publication.

Fig. S1. $B(X)$ versus $X$ (red solid line) calculated using Eq. (S12) and used in this publication. Sofue [[2](#_ENREF_2)] evaluation of $B(X)$ (black dotted line), determined manually from his Fig. 3.

**References**

1 Sofue, Y., Honma, M. & Omodaka, T. Unified rotation curve of the Galaxy - decomposition into de Vaucouleurs bulge, disk, dark halo, and the 9-kpc rotation dip. *Publ. Astron. Soc. Japan* **61**, 227 (2009).

2 Sofue, Y. Grand rotation curve and dark-matter halo in the Milky Way galaxy. *Publ. Astron. Soc. Japan* **64**, 75 (2012).
